# The first complete genome sequence of *Staphylococcus aureus* ST5477

**DOI:** 10.1128/mra.01004-23

**Published:** 2024-01-05

**Authors:** Pimlapas Leekitcharoenphon, Saria Otani, Judit Szarvas, Tutu Mzee, Happiness Kumburu, Frederik Duus Møller, Frank M. Aarestrup

**Affiliations:** 1 Research Group for Genomic Epidemiology, National Food Institute, Technical University of Denmark, Kgs. Lyngby, Denmark; 2 Ifakara Health Institute, Bagamoyo Branch, Bagamoyo, Tanzania; 3 Department of Molecular Biology and Biotechnology, University of Dar es Salaam, Dar es Salaam, Tanzania; 4 Kilimanjaro Clinical Research Institute Institute, Moshi, Kilimanjaro, Tanzania; 5 Kilimanjaro Christian Medical Centre, Moshi, Kilimanjaro, Tanzania; 6 Kilimanjaro Christian Medical University College, Moshi, Kilimanjaro, Tanzania; Rochester Institute of Technology, Rochester, New York, USA

**Keywords:** complete genome, whole genome sequencing, *Staphylococcus aureus*, ST5477

## Abstract

This study presents the first complete genome of *Staphylococcus aureus* ST5477, one of the most common sequence types (ST) from bovine in eastern Africa. The genome consists of a 2,723,132-bp circular chromosome and a 3,044-bp plasmid. This strain was collected in 2017 from cow milk in Tanzania.

## ANNOUNCEMENT

*Staphylococcus aureus* is a public health problem worldwide and an important opportunistic pathogen in both humans and animals (1). *S. aureus* is common in mastitis cases in East African countries (2). Most of the reported *S. aureus* strains in Africa were novel, belonging to CC97 (3, 4). More recent studies have reported that ST5477 is one of the major ST types as bovine adapted *S. aureus* and causes mastitis, with a number of sequenced strains (2,5). Nonetheless, none of them has been made as a complete genome. This study reports the first complete *S. aureus* ST5477.

*S. aureus* ST5477 strain BC00107 was extracted from cow milk in Bagamoyo, Tanzania, on the 10th of October 2017. The milk sample was pre-enriched with buffered peptone water at a 9:1 ratio, and the mixture was incubated for 24 h at 37°C (6). The sample was then cultured aerobically at 35°C for 18–24 h on Columbia 5% Sheep Blood Agar and Mannitol Salt Agar. Identification of *S. aureus* was confirmed by a tube coagulase test (7, 8).

High-molecular-weight DNA was extracted from pure culture using Quick-DNA HMW MagBead Kit (Zymo Research, D6060). The DNA was sequenced on Oxford Nanopore platform GridION for long reads using ligation library protocol with 14 chemistry (SQK-NBD114.96) following the manufacturer’s instructions, without fragmentation or size selection steps (Oxford Nanopore Technology, UK). Libraries were run on the 14 chemistry flowcell R10.4.1 (Oxford Nanopore Technology, UK). The reads were basecalled using Guppy_basecaller and “super high accuracy” function yielding 2,160,000 reads with N50 of 13.8 kb. Separate isolation of genomic DNA and short-read sequencing were previously carried out (5) using QIAamp DNA mini kit (Qiagen GmbH, Germany) and Illumina NextSeq 500 platform with a paired-end 2 × 150 bp protocol, respectively. The raw reads were adapter-trimmed, quality filtered using bbduk (part of the suite bbtools version 36.49) (9). The trimmed raw reads consisted of a total of 4,300,802 paired reads and 603 million bp (~115× coverage) of sequence.

Assembly from Oxford Nanopore was performed using Trycycler version 0.5.4 (10) together with two long-read assemblers, Flye version 2.9.1 (11) and Raven version 1.8.1 (12). Subsequently, the assembly was carried out for small-scale errors such as single base pair substitutions and indels using Illumina reads and Polypolish version 0.5.0 (13). The assembly resulted in a single chromosome (2,723,132 bp, with 33% GC content) and a plasmid (3,044 bp, with 31% GC). The genome was annotated using the NCBI Prokaryotic Genome Annotation Pipeline (PGAP) (14). There were 2,692 genes representing 2,610 protein-coding sequences and 19 rRNAs, 59 tRNAs, 4 ncRNAs, and 117 pseudogenes.

## Data Availability

The genomic data were submitted in GenBank under BioProject accession number PRJEB59926. The chromosomal sequence for *S. aureus* ST5477 str. BC00107 can be obtained at GenBank under the accession number CP135966, and the sequence of plasmid can be found under the accession number CP135967. The Oxford Nanopore reads can be downloaded at The European Nucleotide Archive (ENA) accession number ERR12076611 (ERX11458978). The previously sequenced Illumina sequence can be located at ENA accession number ERR10898915 (ERX10341453).
